# Advances in antitumor research of CA-4 analogs carrying quinoline scaffold

**DOI:** 10.3389/fchem.2022.1040333

**Published:** 2022-10-28

**Authors:** Chao Wang, Jing Chang, Shanbo Yang, Lingyu Shi, Yujing Zhang, Wenjing Liu, Jingsen Meng, Jun Zeng, Renshuai Zhang, Dongming Xing

**Affiliations:** ^1^ Cancer Institute, The Affiliated Hospital of Qingdao University, Qingdao University, Qingdao, China; ^2^ Qingdao Cancer Institute, Qingdao University, Qingdao, China; ^3^ The Affiliated Cardiovascular Hospital of Qingdao University, Qingdao University, Qingdao, China; ^4^ School of Life Sciences, Tsinghua University, Beijing, China

**Keywords:** inhibitor of tubulin polymerization, CA-4, CBSI, quinoline, antitumor

## Abstract

Combretastatin A-4 (CA-4) is a potent inhibitor of tubulin polymerization and a colchicine binding site inhibitor (CBSI). The structure-activity relationship study of CA-4 showed that the *cis* double bond configuration and the 3,4,5-trimethoxy group on the A ring were important factors to maintain the activity of CA-4. Therefore, starting from this condition, chemists modified the double bond and also substituted 3,4,5-trimethoxyphenyl with various heterocycles, resulting in a new generation of CA-4 analogs such as chalcone, Flavonoid derivatives, indole, imidazole, etc. Quinoline derivatives have strong biological activity and have been sought after by major researchers for their antitumor activity in recent years. This article reviews the research progress of novel CA-4 containing quinoline analogs in anti-tumor from 1992 to 2022 and expounds on the pharmacological mechanisms of these effective compounds, including but not limited to apoptosis, cell cycle, tubulin polymerization inhibition, immune Fluorescence experiments, etc., which lay the foundation for the subsequent development of CA-4 containing quinoline analogs for clinical use.

## 1 Introduction

**FIGURE 1:**
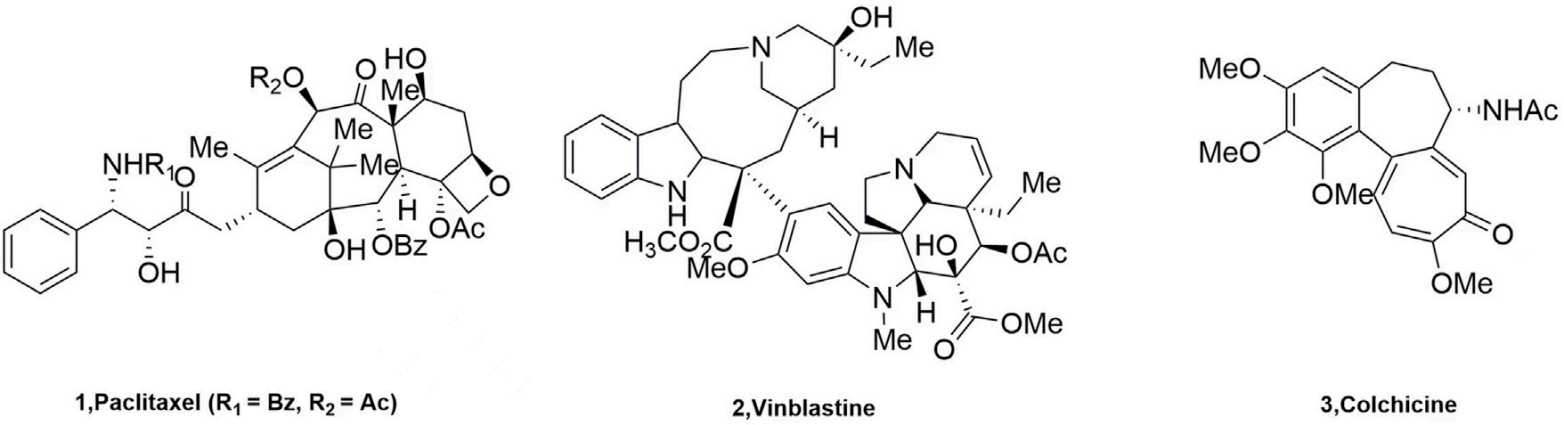
The structure of tubulin inhibitors.

Microtubules, a key building block of the cytoskeleton, are dynamic polymers of tubulin that form an ordered network of polarized tubules (Honore et al., 2005). Microtubules are formed by combining *α* and *β* heterodimers, which are important components of the eukaryotic cytoskeleton, and they have played important roles in mitosis and cytokinesis (Lu et al., 2012). The vast majority of these molecules act by binding to a heterodimer of the protein microtubulin (*α*, *β*) that forms the microtubule core (Wang et al., 2021). The microtubulin heterodimer contains six binding sites (Pryor et al., 2002): Pironetin, Taxane, Laulimalide/Peloruside, Vinca, Maytansine, and Colchicine binding sites. Targeted drugs that act on microtubules and exert anti-tumor effects by inhibiting microtubule proteins are called microtubule protein inhibitors. They were classified into two categories according to their mechanism of action on microtubules: microtubule protein depolymerization inhibitors, such as Paclitaxel (**1**, Figure 1), and microtubule protein polymerization inhibitors, such as Vinblastine (**2**, Figure 1) and Colchicine (**3**, Figure 1).

**FIGURE 2:**
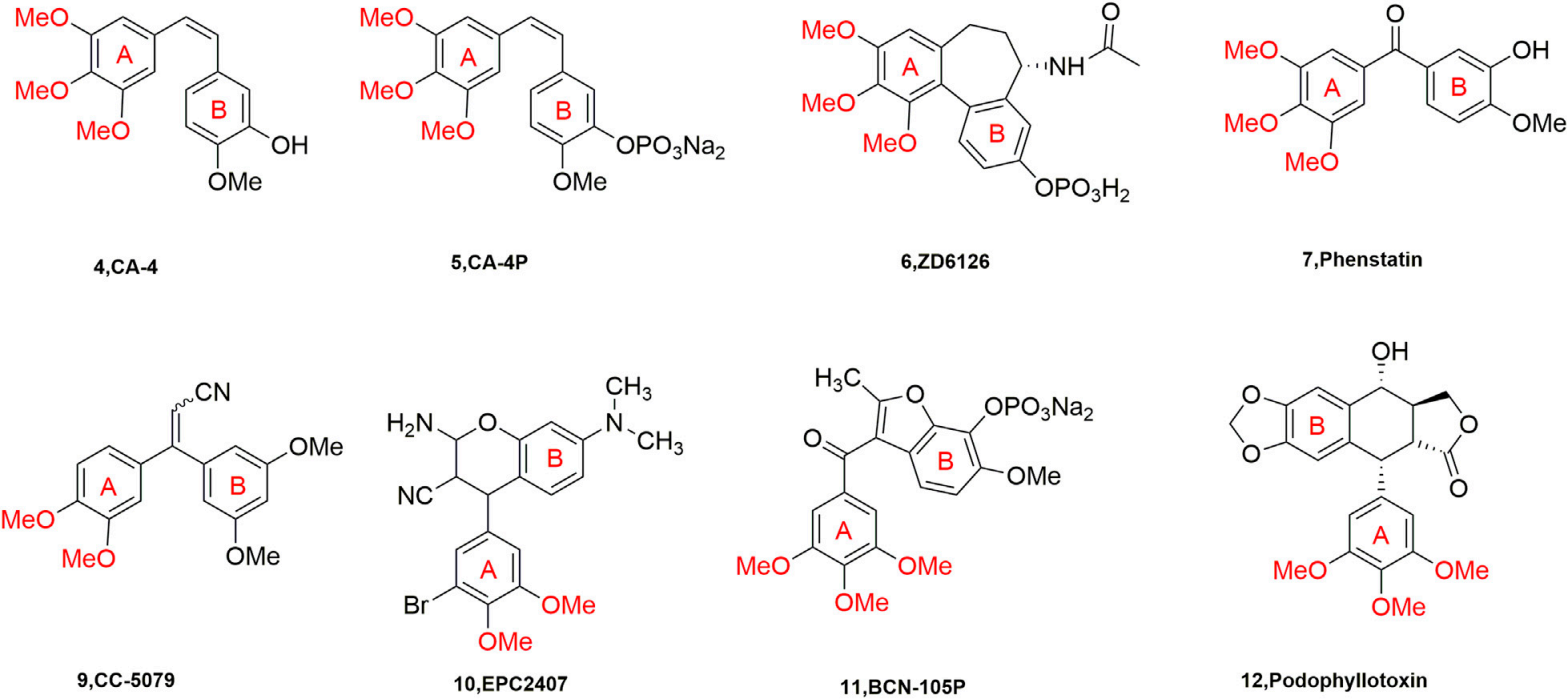
Chemical structures of some CBSIs (currently in clinical trials).

Current studies show that there are many drugs used in clinical oncology that act on the paclitaxel and vincristine binding sites. These drugs are very effective but have complex structures due to their large molecular weights, which makes the major researchers discouraged. However, the compounds acting on CBSI have the characteristics of small molecular weight, simple structure, diverse molecules, and easy synthesis and transformation, which are sought after by major researchers (Gigant et al., 2005). A number of representative tubulin inhibitors (currently in clinical trials) targeting the colchicine binding site have emerged (Figure 2).

**FIGURE 3:**
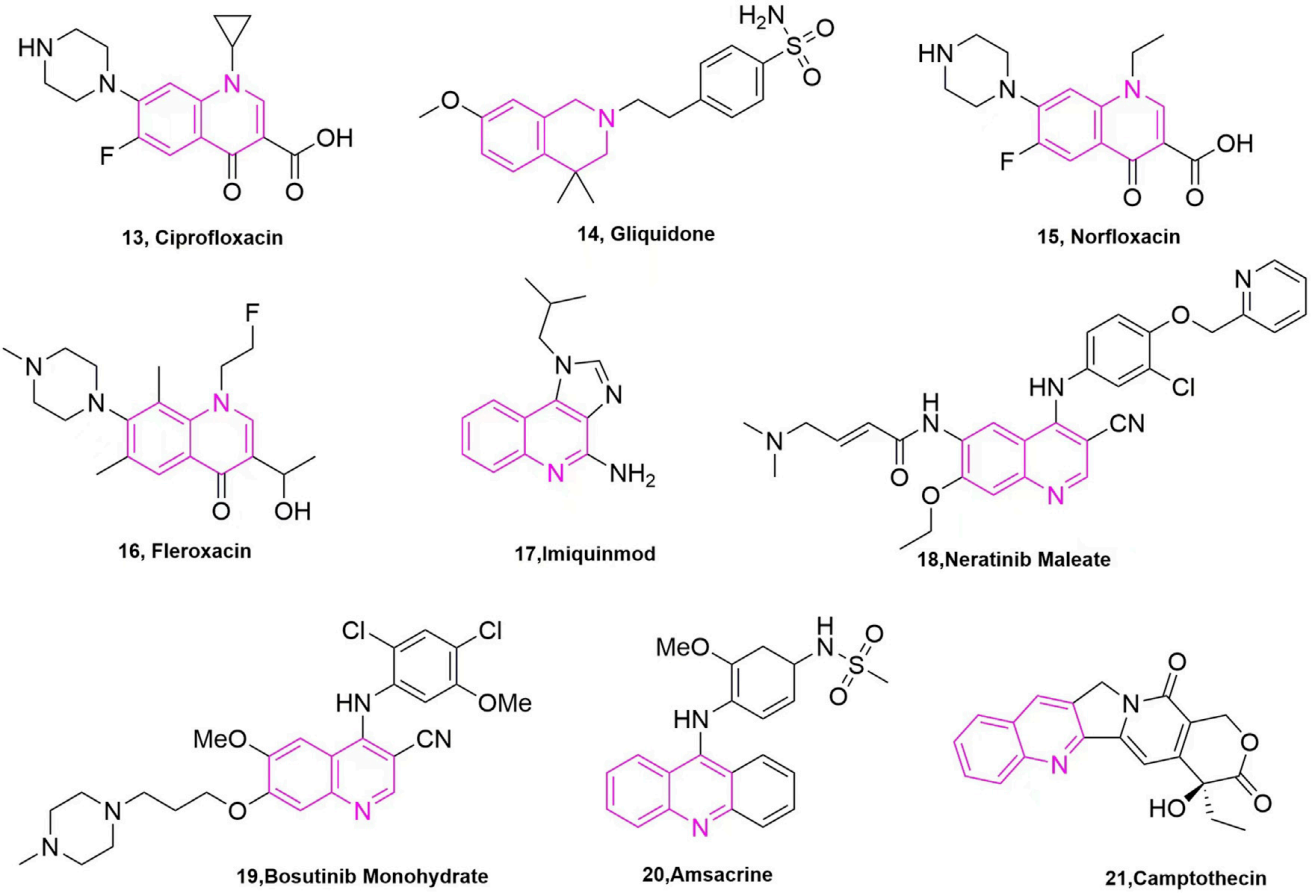
The marketed drugs carrying quinoline’s structure.

It is well known that quinoline was isolated in the 19th century and since then more and more related natural products have been isolated and identified. Derivatives containing quinoline show great advantages due to nitrogen atoms, such as can increase the basicity of molecules, because of its basic properties and the possibility of nitrogen atoms forming strong hydrogen bonds with the target. Another important property is polarity, which can be used as a means to reduce lipophilicity and improve water solubility and oral absorption (Alvarez et al., 2016). Quinoline groups are often present in natural alkaloids with a wide range of biological activities and there are many drugs that carry quinoline on the market (Figure 3). Molecules containing quinoline scaffolds can even enhance the cytotoxicity of doxorubicin against multidrug-resistant cancer cell lines at non-toxic concentrations (Chen et al., 2014). Quinoline analogues have also shown anticancer activity by different mechanisms, including alkylating agents, tyrosine kinase inhibitors, and microtubulin inhibitors (Kaur et al., 2010).

**FIGURE 4:**
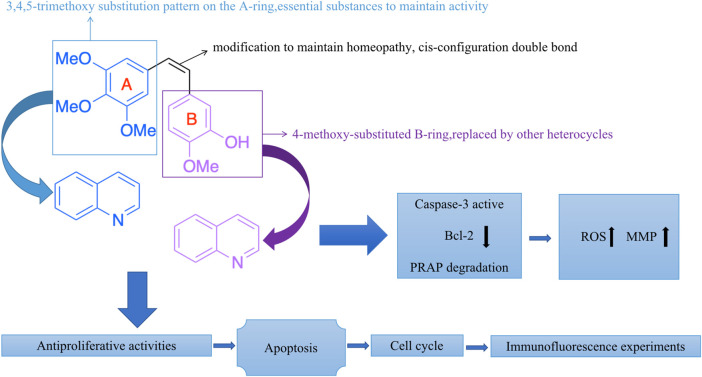
The mechanism of action of these compounds (CA-4 analogs).

**FIGURE 5:**
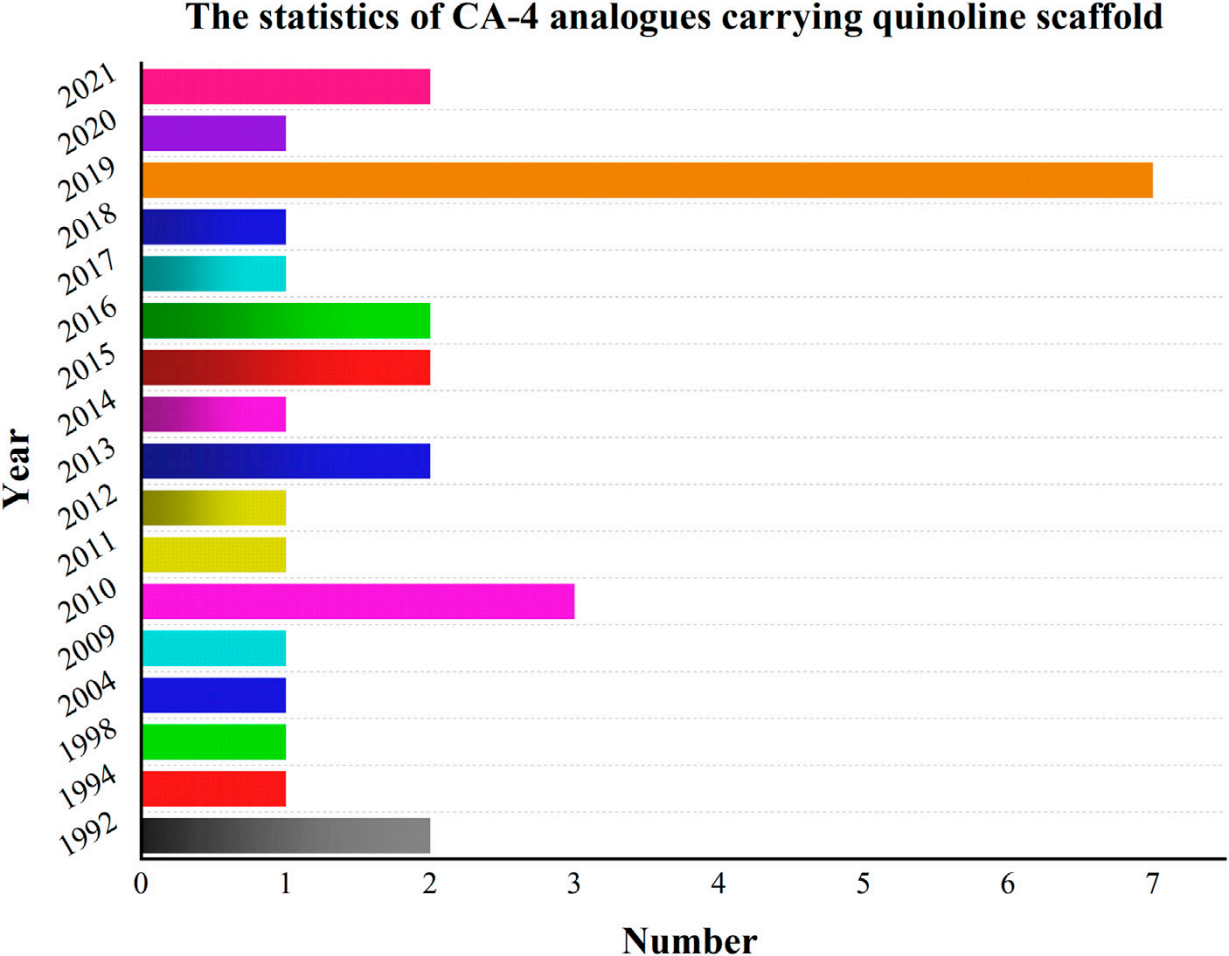
Relevant articles have been published from 1992 to 2022.

Meanwhile, extensive structure-activity relationship (SAR) studies were performed on CA-4, which demonstrated the 3,4,5-trimethoxy substitution pattern on the A-ring, 4-methoxy-substituted B-ring and cis-configuration double bond as the basis for the polymerization-inhibiting activity of microtubulin (Stefanski et al., 2018). We have mapped the mechanism of action of these compounds (CA-4 analogs) based on the SAR of CA-4 (Figure 4). Among the modifications of the CA-4 structure, some researchers have modified it using quinoline and *iso*-quinoline structures. Their study also further demonstrates that the quinoline fraction may be an alternative to the conventional 3,4,5-trimethoxyphenyl fraction when bound to the colchicine site (Li et al., 2019). In addition, the use of other heterocycles to replace the B-ring has been pursued by various researchers (Solomon et al., 2019). They designed and synthesized some derivatives that carry CA-4 quinoline analogues and interact with the colchicine binding site. The results showed that the successfully modified compounds had comparable activity to CA-4, and some had higher activity than CA-4. In addition, we searched PubMed, Web of science and Scoups for 30 related articles published between 1992 and 2022 (Figure 5). This paper mainly reviews the design, synthesis and validation of pharmacological activities of quinoline fragment-containing CA-4 derivatives in these articles, including the experiments involved and the corresponding experimental results. Finally, the similarities and differences among these articles are pointed out, and the outlook is proposed.

## 2 CA-4 analogues carrying quinoline scaffold

### 2.1 Modification of CA-4

#### 2.1.1 Modification of A-ring

**FIGURE 6:**
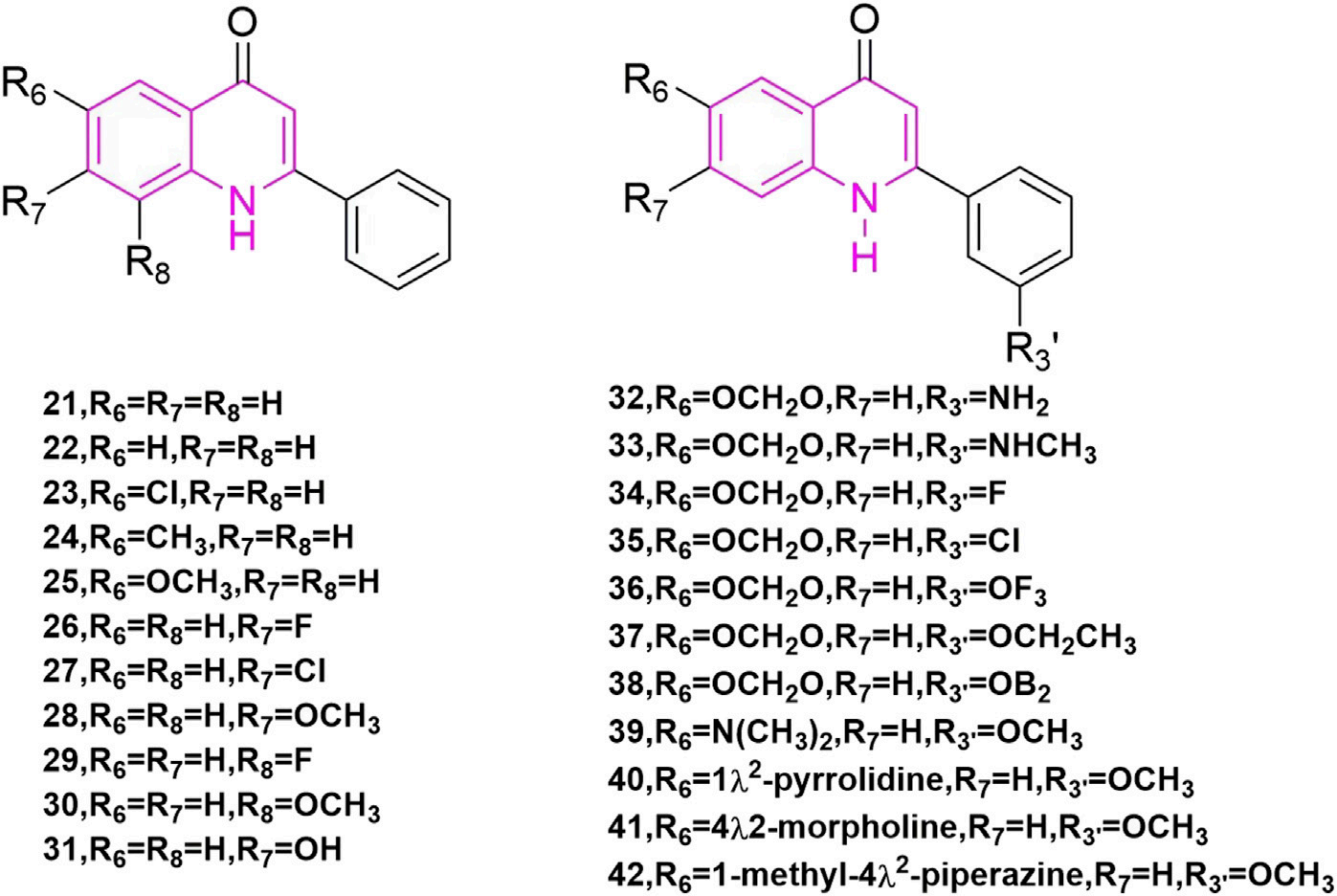
The structures of the compounds designed by Kuo et al. (1993) and Li et al. (1994b).

Kuo et al. (1993) started with a tricyclic chemical structural pattern, which was the first and similar to CA-4 analogues carrying quinoline scaffold. They designed and synthesized a series of 1,6,7,8-substituted 2-(4′-substituted phenyl)-4-quinolones, which had been evaluated as cytotoxic compounds and as antimitotic agents that interact with tubulin. These compounds were assayed for their cytotoxicity *in vitro* against six tumor cell lines, which included human lung carcinoma cell lines (A-549), ileocecal carcinoma cell lines (HCT-8), and melanoma cell lines (RPMI-7951), and epidermoid carcinoma of the nasopharynx cell lines (KB) and two murine leukaemia cell lines (P-388 and L1210). In these compounds, compound **23** (Figure 6), compound **25** (Figure 6), and compound **26** (Figure 6) showed potent cytotoxicity (Monks et al., 1991), with EC_50_ values < 1.0 μg/ml in all cancer lines. They then tested the effects of these three compounds on tubulin interactions, which used the COMPARE algorithm (Bai et al., 1991) assay and compound **25** showed the best with an IC_50_ value of 2.7 ± 0.04 μM. In addition, these compounds could inhibit the binding of radiolabelled colchicine to tubulin, compared to colchicine, podophyllotoxin, and CA-4, and compound **25** showed the best of them. Even so, the three compounds were not as potent as natural products.

Li et al. (1994b) is a continuation of Kuo et al., they began studying 2-phen-yl-quinolones and designing and synthesizing a new series of compounds, which illustrated strong inhibitory effects against a variety of human tumor cell lines, including those derived from solid tumors. Compound **28** (Figure 6) illustrated great activity and also inhibited tubulin polymerization (Li et al., 1994a), with activity comparable to those of the potent antimitotic natural products colchicine, podophyllotoxin, and CA-4. Compounds **21**, **23**, **24**, **27**–**29**, **33**, **40**, and **41** (Figure 6) demonstrated strong cytotoxic effects to most of the tumor cell lines with GI_50_ values in the micromolar to nanomolar range. Also, compounds **42** are just to enrich the structure of quinoline compounds.

**FIGURE 7:**
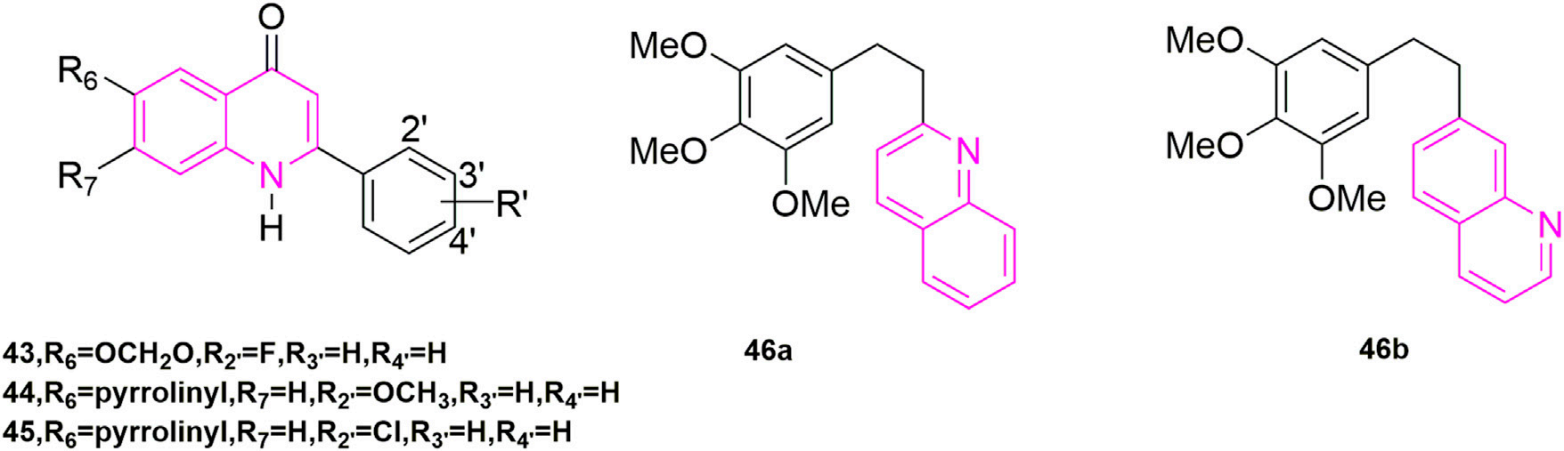
The structures of the compounds designed by Xia et al. (1998) and Perez-Melero et al. (2004).

Xia et al. (1998) designed and synthesized a novel series of 6,7,2′,3′,4-substituted-1,2,3,4-tetrahydro-2-phenyl-4-quinolones based on the structure of natural anti-mitotic drugs. They evaluated cytotoxicity (Rubinstein et al., 1990) in six cancer cell lines, including HCT-8, breast cancer cell lines (MCF-7), A-549, KB, renal cancer cell lines (CAKI-1), and melanoma cancer cell lines (SKMEL-2). In these compounds, compounds **43**, and **44**, **45** (Figure 7) illustrated great cytotoxic effects with ED_50_ values in the nanomolar or sub nanomolar range. Compound **44** demonstrated the best activity, pyrrolidinylrrolinyl ring at the 6-position, similar with Li’s research before.

#### 2.1.2 Modification of B-ring and *cis*-double bond

By studying the conformational relationship of CA-4, Perez-Melero et al. (2004) proposed using 2-naphthyl moiety to replace the 3-hydroxy-4-methoxyphenyl ring of CA-4, which could not observably reduce cytotoxicity and inhibit tubulin polymerization activity. Then they came up with using 6- or 7-quinolyl systems to substitute 3-hydroxy-4-methoxyphenyl ring, keeping the 3,4,5-trimethoxyphenyl moiety as A-ring at the same time (**46a**, **46b**, Figure 7), getting a new family of analogues of CA-4. Compound **46a** was highly cytotoxic but did not inhibit tubulin polymerization. While compound **46b** was similar to the naphthalene-containing compound previously described and had powerful cytotoxicity [−log (IC_50_) M = 6.8] and tubulin inhibition ability. Other compounds demonstrated weak inhibition of tubulin compared to CA-4. In addition, the presence of N atoms also made these compounds more water-soluble than CA-4, which pointed out the way to solve the water-soluble problem of CA-4.

**FIGURE 8:**
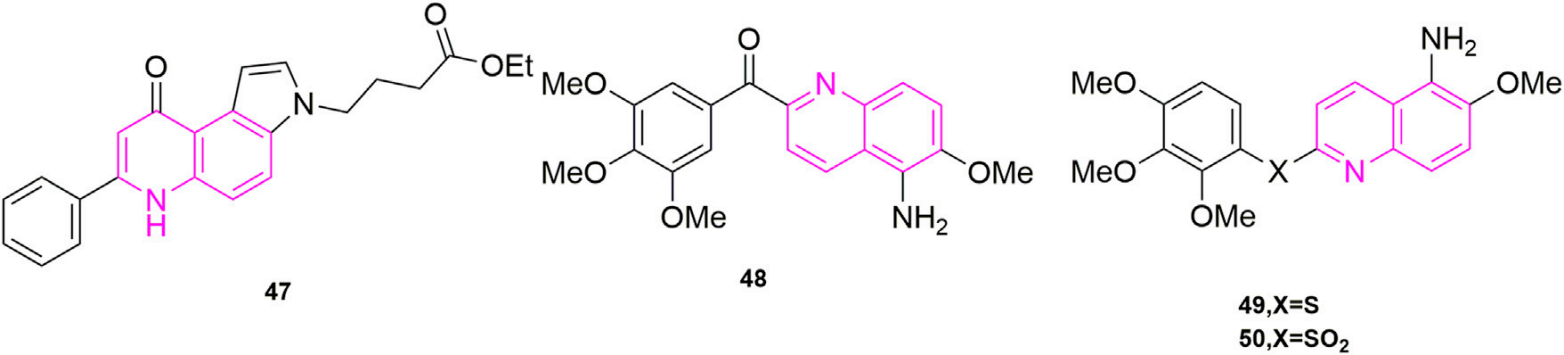
The structures of the compounds designed by Ferlin et al. (2010), Nien et al. (2010), and Lee et al. (2011).

Ferlin et al. (2010) designed and synthesized a series of new substituted 7-phenyl-3*H*-pyrrolo [3,2-f] quinolin-9-ones, which starting from the study of the antiproliferative activity (Gasparotto et al., 2007) of this structure of pyrroloquinoline. By evaluating its antiproliferative activities, the best active compound **47** (Figure 8) was found to be highly selective for human leukaemia cells (GI_50_ = 0.45 ± 0.10 μM), and it also blocked Jurkat cells in the G2/M phase in a concentration-dependent manner. At a concentration of 0.06 μM for 24 h compared to the control the number of G2/M cells increased from 9.4% to 77% in the control group. G1 cells decreased from 47% to 3% in the control group, while S-phase cells decreased from 43% to 20% (Gasparotto et al., 2007). Such results were reflected in the other compounds as well. To verify that subsequent experiments were relevant, apoptosis experiments were conducted, in which they selected Jurkat cells treated with compound **47** for 24 h and 48 h to observe the extent of apoptosis (Martin et al., 1995; Vermes et al., 1995). The effectiveness led them to further investigate the mechanism of apoptosis, so they carried out mitochondrial depolarization experiments and confirmed that compound **47** could dose-dependently increase the mitochondrial transmembrane potential. To investigate the relationship between mitochondrial depolarization and reactive oxygen species, they used compound **47** to treat cells to see if reactive oxygen species production increased, and the results confirmed that it was as expected and induced reactive oxygen species (ROS) production in the cells (Zamzami et al., 1995). This was the same increase in mitochondrial transmembrane potential as in the mitochondrial depolarization assay. Finally, they found that Jurket cells treated with compound **47** activated caspase-3 in a concentration-dependent manner *in vitro*, while an increase in Poly ADP-ribose polymerase (PARP) fragments and a decrease in Bcl-2 protein were also observed in Western blot experiments. In their experiments, compound **47** induced apoptosis by activating caspase-3, cleavage PARP, and downregulating Bcl-2 protein (Earnshaw et al., 1999; Denault and Salvesen, 2002).

Nien et al. (2010) designed and synthesized a series of new aryl quinoline derivatives by coupling the quinoline nucleus with 3,4,5-trimethoxybenzoyl from the pharmacological activity of the parent quinoline nucleus and combining it with CA-4 conformational relationship. *In vitro*, antiproliferative activityexperiments were also performed, and five cells were selected, including KB, non-small-cell lung carcinoma cell lines (H460), human colorectal carcinoma cell lines (HT29), and human gastric cancer cell lines (MKN45), as well as the MDR-positive cancer cell lines, KB-vin10 cells, that overexpressed P-GP 170/MDR. Compound **48** (Figure 8), which contained 5-amino-2-aroylquinolines, was shown to be the most potent of the five cells (IC_50_ = 0.2–0.7 nM), surpassing CA-4. To further understand its potentially active structure, molecular docking was performed, and the results showed that the presence of hydrogen bonds increased the potential of compound **48** and contributed to its effectiveness. To investigate the anti-microtubule protein activity and the ability of compound 36 to compete for colchicine binding sites (Kuo et al., 2004), CA-4 was selected as a control, and compound **48** was effective in inhibiting microtubule aggregation (IC_50_ = 1.6 μM) compared to CA-4 (IC_50_ = 2.1 μM), which was positively correlated with its anti-proliferative activity. The results of the [^3^H]-colchicine binding assay showed that compound **48** bound tightly to the colchicine binding domain with a binding affinity comparable to that of CA-4.

**FIGURE 9:**
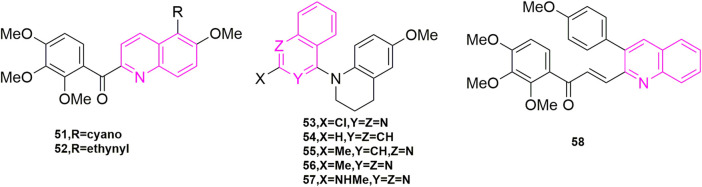
The structures of the compounds designed by Lee et al. (2012), Wang et al. (2013b), and Tseng et al. (2015).

Lee et al. (2011) used amine, sulphide, and sulfone group substitution of compound carbonyls from compound **48** (Figure 8) based on Nien’s study. The results showed that compounds **49** (mean IC_50_ = 42 nM) and **50** (mean IC_50_ = 12 nM) (Figure 9) containing sulfide and sulfone groups between 3,4,5-trimethoxyphenyl ring and 5-amino-6-methoxyquinoline showed significant antiproliferative activity against KB, HT29 and MKN45 cells. Compound **49** inhibited microtubulin polymerization (Kuo et al., 2004; Liou et al., 2004) (IC_50_ = 2.0 μM), similar to CA-4. They went on to study the C-5 substituent of the 3′,4′,5′-trimethoxybenzoyl-6-methoxyquinoline derivative and confirmed that compound **49** containing the hydroxyl group had excellent antiproliferative activity (IC_50_ = 3.4 nM) and microtubule-stabilizing potency of 1.5 μM, greater than that of CA-4 (IC_50_ = 1.9 μM). It also blocked blood vessels and when HUVECs were treated with compound **49** at 15, 30, 60, and 120 nM, compound **49** was able to destroy the formed capillaries in a concentration-dependent manner. In addition, compounds **49** and **50** showed significant efficacy against MDR/MRP-related drug-resistant cell lines (KB-vin10, KB-S1, and KB-7D) with mean IC_50_ values of 2.6–6.7 nM, respectively.

Lee et al. (2012) continued his previous studies, also starting with compound **49**, and continued to investigate the C-5 substituents of 3,4′,5′-trimethoxybenzoyl-6-methoxyquinoline derivatives, choosing cyano, acetylenic and various aryl groups that are less used by major chemists to design and synthesize a new series of new aryl quinoline derivatives. Three human cancer cell lines, KB, HT29, and MKN45 cells were assayed for antiproliferative activity. Compound **51** and compound **52** (Figure 9) exhibited significant antiproliferative activity with mean IC_50_ values of 30 and 57 nM, comparing to colchicine. Interestingly, when they attempted to validate the anti-microtubulin activity and colchicine binding activity of compounds **51** and **52** using colchicine and CA-4 as controls, the binding affinity of cyano **51** and alkyne **52** to the colchicine binding site (Nien et al., 2010) was introduced at 47% and 44% respectively with a 2-fold reduction in activity compared to CA-4, suggesting that both compounds may be bound to another binding site of colchicine binding. In addition, compound **51** and compound **52** were selected to observe the anti-microtubulin activity at concentrations of 1.25, 2.5, 5, 10, and 20 μM, respectively, and showed complete inhibition of microtubulin activity at a concentration of 10 μM. Then they each also inhibited MAP-rich microtubulin aggregation in a concentration-dependent manner. Finally, the reason whether the introduction of cyano and alkyne groups is related to the mode of microtubule protein interactions is unclear at present.

Wang et al. (2013b) also continued his previous research by exploring three design options using methyl 6-chloro-2-(N-(4-methoxyphenyl)-N-methyl) amino nicotinate as the lead compound. The first was that they wanted to explore how to affect the growth inhibitory activity of tumor cells through some specific modifications, the second was that they opted for a conformational restriction strategy that explored feasible binding conformations and possible ligand-target interactions, and the third was to enhance the affinity to the binding site on microtubule proteins to produce good antitumor activity. A total of 24 new compounds were designed and synthesized using these three strategies, of which compound **53** (Figure 9) was the most active and showed high cytotoxicity (Perez-Sayans et al., 2010; Hung et al., 2012) (GI_50_ = 1.5–1.7 nM), especially against P-gp-expressing multidrug-resistant cell lines (vincristine-resistant KB) (KB-vin), in antiproliferative activity against four human tumor cell lines [A549, KB, KB-vin, and prostate cancer (DU145)] using paclitaxel as control (GI_50_). Analogues **54**, **55**, **56**, and **57** (Figure 9) were also comparable [GI_50_ = (0.011–0.19 μM)]. In further studies, the active compounds **55**–**57** significantly inhibited microtubulin polymerization (IC_50_ = 0.92–1.0 μM) and strongly inhibited colchicine binding to microtubulin (75%–99%), using CA-4 (IC_50_ = 0.96 μM) as a control. In contrast to the previously designed pharmacological mechanism studies, they involved the study of drug solubility (Log P parameter) and *in vivo* metabolic stability (Roehm et al., 1991; Sun et al., 2012), in which five compounds, compounds **54**, **55**, **56** (3.21–7.67 μg/ml) exhibited better water solubility than the other two compounds (1.0 μg/ml), while with propranolol (t_1/2_ = 3–5 h) and terfenadine (t_1/2_ = 3 h) were used as positive controls to further assess the metabolic stability of compounds **54–56** by *in vitro* human liver microsomal culture assays. Compound **54** (t_1/2_ = 7.89 min) and compound **53** (t_1/2_ = 10.59 min) were unstable and rapid metabolism may help to reduce drug toxicity *in vivo* and could be used as novel anticancer candidates.

Tseng et al. (2015) designed and synthesized a series of novel 3-phenylquinolinone derivatives against breast cancer and performed antiproliferative activities *in vitro* on three breast cancer cell lines (MCF-7, MDA-MB-231, and SKBR-3) and one non-cancerous normal epithelial cell line (H184B5F5/M10). Among them, (*E*)-3-[3-(4-methoxyphenyl) qui-nolin-2-yl]-1-(3,4,5-trimethoxyphenyl) prop-2-en-1-one (compound **58**, Figure 9) showed anti-proliferative activity against MCF-7 (IC_50_ = 1.05 μM), MDA-MB-231 (IC_50_ = 0.75 μM) and SKBR-3 (IC_50_ = 0.78 μM) with growth inhibitory activity and no significant cytotoxicity against normal H184B5F5/M10 cell lines (IC_50_ > 10 μM). Therefore, a series of pharmacological mechanism studies were subsequently performed. MDA-MB-231 cells were selected for cell cycle experiments and compound **58** at 1, 5, and 10 μM concentrations, with CIL-102 used as a positive control, were treated together for 12, 24, and 36 h. As shown, 28% and 36% of cells were arrested in the G2/M phase when treated with 1 μM and 5 μM compound **58**, respectively, indicating that the induced cell cycle was concentration and time-dependent. Western blot experiments showed, downregulation of CDC25c, CDK1, and the cell cycle protein cyclin B1 in cells treated with compound **58**. To further investigate the mechanism, they selected CLI-102 as a control (10 μM) and selected the same concentration of compound **58** as in the cell cycle experiments (1, 5, and 10 μM), performed immunofluorescence experiments, and they found that the cells exhibited filamentous structures and a reduced round tube state in the cytoplasm. In addition, they went on to perform Western blot experiments to observe the microtubulin polymerization inhibition assay, and as shown, the microtubulin polymer form showed a concentration-dependent decrease, indication inhibition of microtubulin polymerization. They concluded that the inhibition of cell growth by compound **58** was due to apoptosis and they analysed the effect of compound **58** on the anti-apoptotic protein Bcl-2 and the pro-apoptotic proteins Bad and Bax. Compound **58** had no significant effect on the expression of apoptotic protein Bad, but upregulated apoptotic protein Bax and downregulated anti-apoptotic protein Bcl-2. In parallel they also evaluated caspase-3, caspase-8 and PARP in compound **58** treated MDA-MB-231 cells by Western blot assay. From this experiment they confirmed that compound **58** induces cell cycle arrest in the G2/M phase of PARP by activating caspase-3, caspase-8, which leads to cell death.

Chaudhary et al. (2016) started with the structure of CA-4 to ensure that the A and B ring remain *Z*-form. Like other chemists, they also considered various linkers to replace the double bond. Among their considerations, they selected the 2-aminoimidazole backbone, a backbone that is also a valuable molecular motif in medicinal chemistry. They designed and synthesized a series of 4,5-diaryl-2-aminoimidazoles and in performing experimental studies for antiproliferative activities, they found that compound **59** (Figure 12) showed good activity against five types of human cancer cells, which are MCF-7 (IC_50_ = 3 ± 2 nM), HeLa (IC_50_ = 10 ± 1 nM), hepatocellular carcinoma (HuH-7, IC_50_ = 335 ± 10 nM), MDA-MB-231(IC_50_ = 96 ± 13 nM) and drug-resistant mouse breast cancer (EMT6/AR1, IC_50_ = 350 ± 7 nM). In these cells, compound **59** was more potent than CA-4 (IC_50_ = 18 ± 2, 25 ± 2, 430 ± 9, 332 ± 32, and 495 ± 11 nM), and they speculated that it might be that the quinoline ring played a role in addition to the 2-aminoimidazole backbone. It might be due to the biological target—the N-/NH and C2-amine functional groups of the 2-aminoimidazole and quinoline N rings in the ligand play the role of hydrogen bonding to stabilize the ligand. Immunofluorescence experiments were performed immediately after and they found that compound **59** mildly depolymerized microtubules at a concentration of 5 nM and strongly depolymerized MCF-7 cells at 10 nM, which found that the compound blocked the single-stage spindle in mitosis. Meanwhile, they performed cell cycle experiments using MCF-7 cells using CA-4 as a control, and 40% and 74% of the cells were in the G2/M phase at 5 and 10 nM of compound **59** concentrations, which compared to CA-4 (20% and 60%). To verify the reliability of the results, they stained the cells with Ser-10, which showed a concentration-dependent increase in the number of staining group proteins compared to the carrier cells. They evaluated the effect of compound **59** on MDA-MB-231 wound healing in migrating tumour cells, using CA-4, a well-known antivascular agent, as an example. Cells in the Control group had complete wound closure after 18 h. The results showed that compound **59** strongly inhibited the migration of MDA-MB-231 (50 and 150 nM, 56% ± 3% and 52% ± 4% inhibition) cells compared to CA-4 (50 nM, 8% ± 1% inhibition). Finally, they verified that the compound **59** is bound to the colchicine binding site (Cortese et al., 1977; Sackett, 1993) of microtubulin by measuring the fluorescence intensity of compound **59**, selected from CA-4 mycotoxin, paclitaxel, and vincristine.

**FIGURE 10:**
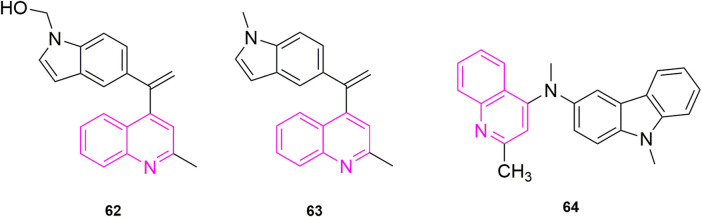
The structures of the compounds designed by Li et al. (2019) and Khelifi et al. (2017).

Kumari et al. (2019) wanted to further elucidate the specific molecular mechanism and antitumor activity of compound **59** (Figure 10) based on the previous study by Chaudhary. Fibroblasts (L929) and non-tumorigenic mammary epithelial cells (MCF-10A) with IC_50_ values of 147 ± 45, 136 ± 30, and 3,316 ± 493 nM, respectively. In addition, they performed EB1 gene transfection and EB1 comet number calculations and they found that compound **59** strongly depolymerized microtubules of HeLa cells in interphase and mitotic cells, and also observed that reduction in the number of comets in cells treated with 5, 10, and 20 nM compound **59**. To further clarify the mechanism, they investigated the binding of compound **59** to EB1 with microtubules *in vitro*, and the results were due to a reduction in the number of comet cells due to a reduction in the number of microtubules in the cell body, not due to a reduction in EB1 binding to intracellular microtubules. They concluded not only that compound **59** inhibited intermediate microtubule dynamics in HeLa cells, but also that it could also interfere with mitotic spindle dynamics in the cells. They also evaluated chromosomes by examining the effect of compound **59** on chromosome movement by time-lapse imaging of live HeLa cells expressing histone 2B, using cells treated with a concentration of 10 and 20 nM, which showed that the compound **59** inhibited chromosome movement and that these chromosomes could not be aligned on tumid phase plate, and even an increase in chromosome consistency index (CI) was also observed as well as mitotic defects and chromosome misalignment, which could be responsible for the diminished microtubule dynamics. Subsequently, to investigate whether misaligned chromosomes activate the spindle assembly checkpoint (SAC), SAC activation was next examined after compound **59** treatment, which showed that cells treated with compound **59** were blocked at mid mitosis and were unable to enter the ate phase due to SAC activation, and in addition, they observed that compound **59** inhibited mitotic spindle assembly and induced multistage spindle formation. It is known that there are two ways of SAC activation, one is cell slippage through mitosis and the other is apoptosis. To confirm apoptosis, they did Western blot experiments with HeLa cell-associated proteins and showed that compound **59** can activate caspase-3 and caspase-9 to induce cleavage of PREP-1, which is consistent with the previously observed apoptosis. In addition, they also did mitochondrial membrane potential experiments and found that the compound could cause apoptosis by causing a decrease in mitochondrial membrane potential. These two forms of apoptosis will be of further value for subsequent studies. Finally, they were also the third study to perform animal experiments. They used NOD-SCID mice and according to Chaudhary’s study, which used compound **59** and selected MCF-7 cells to determine the effect of xenografts in mice. The results of the study showed 49% reduction in tumor volume in mice treated with compound **59** compared to the control group, with no significant effect on body weight, suggesting a lesser side effect and a worthwhile candidate to investigate for the treatment of cancer.

**FIGURE 11:**
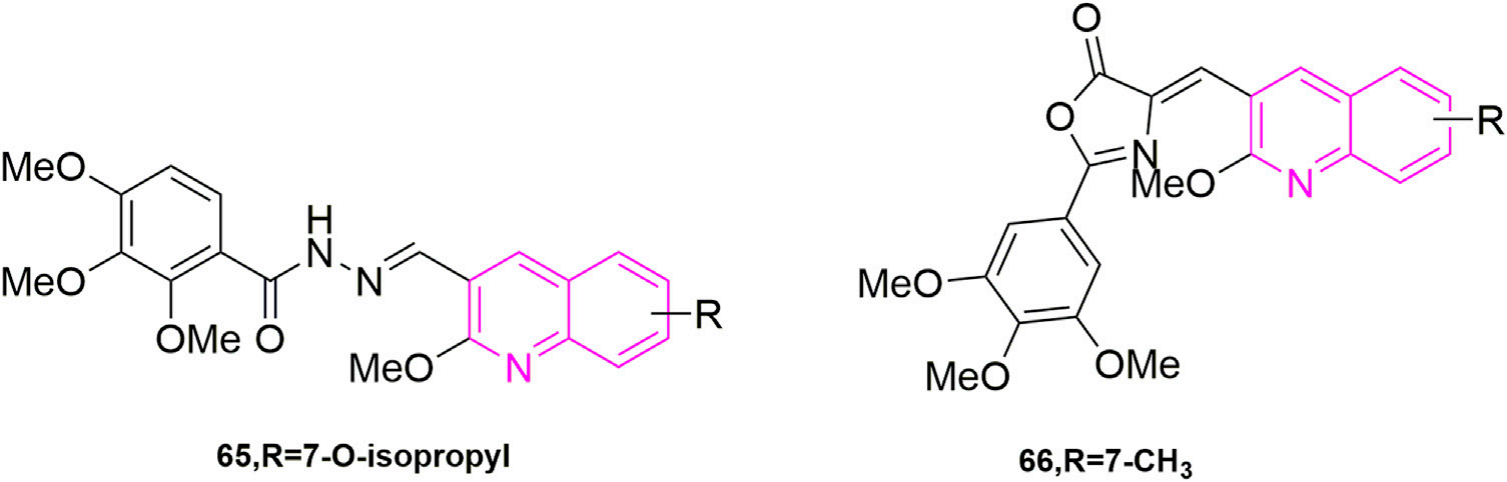
The structures of the compounds designed by Ibrahim et al. (2020, 2021).

Ibrahim et al. (2020) started from the analysis of the structure of CA-4 and proposed the same idea as major chemists to use heterocycles to replace the unstable double-built structure in CA-4, for example. Some chemists used quinoline to replace the B ring showing compounds with 10 times more powerful activity than CA-4. So, they designed two models of compounds, the first model was synthesized using the 3,4,5-trimethoxyphenyl fraction and the anthraquinone fraction as various electronic substituents for bioelectronic isomeric substitution of the B ring of CA-4. The second model is to add structural rigidity by introducing a C-ring (a hydrazone open linker and its cyclic form) to replace the carbene bond, and this prevents CA-4 from isomerizing *in vivo*. These compounds were subsequently tested for anti-value-added activity in four human cancer cells: MCF-7, human leukaemia cells (HL-60), HCT-116, and HeLa. Most of the compounds showed significant antiproliferative activity in the sub-micromolar range, with the most potent compound being compound **65** (Figure 11) (7-tert-butyl-substituted quinoline) (IC_50_ = 0.02–0.04 μM), and they later did antiproliferative activity assays (IC_50_ > 35 μM) in MCF-10A, showing that the compound against MCF-7 cells with good selectivity. Then they further did *in vitro* microtubulin polymerization inhibition assay and colchicine binding site assay, and the results were as expected, compound **65** effectively inhibited microtubulin polymerization and inhibited the colchicine binding site. With the results from the previous step, they continued with the cell cycle assay, selecting compound **65** at a concentration of 50 and 250 nM to treat MCF-7 cells, and the percentage of cells in the G2/M phase increased to 23.7% and 35.6% within 48 h compared to the control, a trend that further increased as time progressed. It indicates that compound **65** can induce MCF-7 cells blocked in the G2/M phase in a concentration-dependent manner. At the same time, they also did an apoptosis assay and MCF-7 cells treated with 50 nM and 250 nM significantly increased to 15% and 29% compared to the control (0.8%), indicating that the sub-compound can induce apoptosis in a concentration-dependent manner. The above results make compound **65** worthies of further investigation as an effective chemotherapeutic agent for targeting microtubulin.

Another study was carried out by Ibrahim et al. (2021), who further optimized CA-4 from its structure by inserting rigid oxazolones and imidazolines between the A and B ring to maintain *cis*-activity, and then maintained 3,4,5-trimethoxyphenyl by altering the electron group substitution effect. The designed compounds were subjected to antiproliferative activity assays against four MCF-7, HL-60, HCT-116, HeLa) using CA-4 as a control, where compound **66** (Figure 11) stood out with the optimal effect (IC_50_ = 0.019–0.042 μM), and more excitingly the compound was selective for cancer cells by verifying that MCF-10A (IC_50_ > 50 μM), compared to an IC_50_ value of 6.1 μM for CA-4. They have since performed more detailed mechanistic studies, such as the next microtubulin polymerization inhibition assay, where they used CA-4 as a control (IC_50_ = 2.17 μM) and found that compound **66** (IC_50_ = 1.21 μM) strongly inhibited microtubulin polymerization. They then proceeded to use compound **66** concentrations at 1 and 10 μM on the ability of colchicine to compete with microtubulin, using CA-4 as a control (86% and 97%), with 79% and 87% inhibition of compound **66**, this result suggests that the odd compound is involved in microtubulin polymerization inhibition *via* the colchicine binding site. After that they continued with cell cycle experiments, wanting to understand the potential anti-value-added activity of compound **66**, and selected 50 and 250 nM to observe the extent of cell block in the G2/M phase at 0, 24, 48, and 72 h. After 48 h, the percentage of the G2/M phase was 28.4% and 38.3% compared to the control (9.2%). After 72 h it was 33% and 40.8%. In addition, they found that the compound also induced phase apoptosis, they also looked at the percentage of apoptosis in cells at 24, 48, and 72 h. They mainly looked at apoptosis at 0 and 250 nM and CA-4 at 50 nM with an increase of 15%, 21%, and 29% respectively, in that order, compared to the control (1%). They then performed Western blot experiments and confirmed that the compound triggered apoptosis in MCF-7 cells by down-regulating the expression level of the anti-apoptotic protein Bcl-2 and up-regulating the expression of the pro-apoptotic protein Bax, while activating caspase-9 and could participate in the mitochondrial apoptotic pathway, causing changes in mitochondrial membrane potential. The final cell scratching assay also confirmed that the compound could effectively inhibit cancer cell migration. In conclusion, this compound **66** is a drug worthy of further investigation for development as a microtubulin inhibitor.

### 2.2 Modification of *iso*-CA-4

**FIGURE 12:**
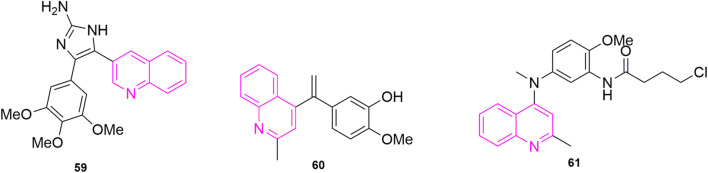
The structures of the compounds designed by Chaudhary et al. (2016), Khelifi et al. (2017), and Tseng et al. (2015).

Khelifi et al. (2017) study found that compounds using a quinoline ring to replace *iso*-CA-4 and 3,4,5-trimethoxy (A ring) in CA-4, while containing 3-hydroxy-4-methoxy on the B ring were the best actor. They tested five human cancer cell lines (IC_50_ < 10 nM), namely human astrocytoma cell lines (U87), acute phase chronic myeloid leukaemia cell lines (K562), Adriamycin-resistant K562 (K562R), A-549 and human colon cancer cells (HCT-116). To further investigate the mechanism, they continued to do immunofluorescence experiments using A549 cells, after treating the cells with compound **60** (Figure 12) for 24 h, mitotic spindle formation was disrupted at 1 nM concentration, and at 5 nM concentration, the microtubule system had been severely disrupted and even multinucleated cells appeared. They performed further cell cycle experiments, selecting compound **60** at concentrations of 0.5, 1, 5, and 10 nM, using dimethyl sulfoxide (DMSO) as a control. It can be observed that the compound **60** blocked the A549 cells in the G2/M phase in a concentration-dependent manner, with a 10 nM concentration blocking the entire population of A549 in G2/M phase (Provot et al., 2013). The final molecular docking showed that when compound **60** was bound to microtubulin, the nitrogen atom of the quinoline A ring could form a hydrogen bond with the Cys241 residue of the B subunit of microtubulin, which was noted that the colchicine binding site to microtubulin showed a binding pattern comparable to that previously used with the quinazoline analogues (*iso*-CoQ) and *iso*-CA-4.

Zhou et al. (2017) resynthesized a series of novel 4-anilinoquinoline derivatives based on their original synthesis of 4-anilino cyclic coumarin derivatives by replacing the coumarin ring with a quinoline ring and performed antiproliferative activity (Sri Ramya et al., 2017) studies in the human colon, lung, ovarian and breast cancer cells, where compound **61** (Figure 12) exhibited potent cytotoxicity (IC_50_ = 1.5–3.9 nM). In addition, they performed cell cycle assays, immunofluorescence assays, microtubule kinetic analysis and colchicine binding site competition assays sequentially to verify the pharmacological mechanism of the antitumor activity of the compound, which were also the first to study the antitumor activity of quinoline analogues in animal models. In the cell cycle assay, compound **61** was selected at a concentration of 3, 10, and 30 nM, the cells present in the G2/M phase (Wang et al., 2015) increased from 15.34% to 80.67%. In immunofluorescence experiments, they chose colchicine and paclitaxel as controls and treated with human ovarian cancer (A27280) cells with 5 nM and 50 nM of compound **61**, and it was seen that the compound **61** significantly disrupted microtubule formation at 50 nM, which was similar to the colchicine effect and even more targeted to microtubule proteins. In the microtubule kinetic analysis assay, compound **61** was taken at a concentration of 0.4, 2, and 10 μM and the control sample was chosen to be colchicine. Compound **61** inhibited microtubule protein polymerizations in a concentration-dependent manner, at 10 μM being significantly stronger than colchicine. In the colchicine binding site (Fortin et al., 2010) competition assay, compound **61** was selected at a concentration of 1, 5, and 25 μM, in Human hepatocellular carcinomas cell lines (HepG2), a dose-dependent inhibition of β-duct formation, leading to the disappearance of the adduct bands, which could be observed by Western blot experiments. Finally, they established an HCT-116 xenograft model in nude mice and selected 5-fluorouracil as a positive control drug (Wang et al., 2013a). They found that compound **61** significantly inhibited tumor growth, with an average inhibition rate of 54.3% already at day 18. The above results laid the clinical foundation for compound **61** to become an anti-tumor drug.

Starting from the structure of *iso*-CA-4, Li et al. (2019) designed and synthesized a new series of quinoline-indole derivatives by replacing the 3,4,5- trimethoxyphenyl and isovanillin in the structure with quinoline and indole rings, respectively, and selected five human cancer cells for evaluation of antiproliferative activity, which were HepG2, KB, HCT-8, MDA-MB-231, and mouse hepatocellular carcinoma cells (H22). The best activities were compound **62** (Figure 10) and compound **63** (Figure 10) (IC_50_ = 2 nM–11 nM), which were more active than CA-4 (IC_50_ = 11 nM–14 nM). To elucidate whether compound **63**, the best active compound, targeted the microtubule protein-microscopic system, they went on to evaluate the effect of the compound on microtubule dynamics in K562 cells, using paclitaxel and colchicine as control, which found that compound 50 had 90.5% binding to colchicine at 5 μM. Further, they evaluated whether the compound **63** can disrupt the cell cycle distribution performed cell cycle experiments, using DMSO as control, the cell stays in G2/M phase increased to 23.21% compared to the control (10.51%). While they performed apoptosis experiments, selected compound **63** concentration at 1, 2, and 4 nM concentration, which was the first time in quinoline analogues to use Flow cytometry to analyse the article of apoptosis. The result that the percentage of apoptotic cells in the control group was 5.96% and after 48 h the total number of apoptotic cells reached 25.7%, 54.9%, and 72.8% respectively, indicating that compound **63** induced apoptosis in K562 cells in a concentration-dependent manner. Furthermore, to further understand whether the compound-induced apoptosis was involved in mitochondrial membrane integrity disruption, JC-1 assay was performed. Again, the concentration of compound **63** was selected as 1, 2, and 4 nM concentration and it was observed from the result that the collapse of mitochondrial membrane potential increased from 6.17% to 28.99%, 45.10%, and 73.55% respectively. This confirmed that apoptosis could disrupt mitochondrial membrane potential integrity. Later they performed immunofluorescence analysis to investigate whether the compound could disrupt microtubule dynamics in the cells, and after treatment of K562 cells with compound **62** at a concentration of 1, 2, and 4 nM for 24 h, which was evident that the microtubule network in the cells was disrupted, and these results indicated that compound **62** could disrupt the microtubule network. Based on the unique anti-vascular activity of CA-4, they then evaluated the anti-vascular activity of compound **62** in human umbilical vein endothelial cells (HUVEC) by performing cell scratching experiments, which could observe that compound **62** could inhibit HUVEC cord formation in a concentration-dependent manner. They designed the compound **62** as the second person to perform animal experiments in quinoline compound design, and they evaluated the antitumor activity of compound **62** and compound **63** by inoculating H22 cells into the right side of mice to establish a mouse model of hepatocellular carcinoma xenograft. They chose paclitaxel, CA-4, and CA-4P as positive controls, and both compounds at a dose of 20 mg/kg/day showed 57.3% and 63.7% tumor reduction at 21 days after treatment, even more than CA-4 (51%) and CA-4P (62.7%), and neither compound **62** and **63** had a significant effect on the body weight of the mice compared to the control drug.

Khelifi et al. (2019) based on the previous design of replacing the 3,4,5-trimethoxyphenyl in CA-4 or *iso*-CA-4 with a quinoline or quinazoline ring to maintain good antitumor activity, designed a new series of compounds based on this by replacing the B ring with a carbazole or indole ring, and they first performed antiproliferative activity assay, in which compound **64** (Figure 10) showed the strongest activity (IC_50_ = 70 pM). Thereafter, antiproliferative activity assays were performed in six more cells, which were A2780, cisplatin-resistant human ovarian tumor (A2780R), human pancreatic cancer (MiaPaca2), K562R, K562R and breast cancer cells (JIM-T1) continued evaluation, which found that the activity of compound **64** was all located within sub-nanomolar levels. Thereafter they performed cell cycle experiments and selected compound **64** at concentrations of 1/5/10 nM, compared to the control DMSO (23%), which blocked the HCT-116 cell cycle at G2/M by 25%, 80%, and 82%, respectively. Somewhat similar to previous methods, they assessed the ability of compound **64** to induce apoptosis by a specific apoptosis assay that cleaved pro-cysteine aspartate to active cysteine aspartate. HCT-116 cells were incubated with 0.5, 1, 5, and 10 nm of compound **64** for 24 h. The activity of caspase-3 and caspase-7 was assessed using a standard cysteines assay, using DMSO as a control. The proteolytic activity of cystoplasties was significantly increased in HCT-116 cells treated with compound **64**, implying that compound **64** induced apoptosis at a low concentration of 5 nM. Finally, they did *in vitro* antiproliferative activity assays, in which flattened endothelial cell aggregates form a reticular vascular network consisting of capillary-like vessels when grown on a stromal gel. After 2, 3, and 5 h, compound **64** was added to the tubular structure at a concentration of 10 nM, and it was observed that compound **64** rapidly disrupted the integrity of the vascular network. Their results also revealed a highly cytotoxic HUVEC showing antiproliferative activity at extended times (72 h) with a GI_50_ value of 3.23 ± 0.28 nM. This did not correlate with the disruption of the vasculature observed in its short time, suggesting that this compound is expected to be further screened as an anti-vascular active drug.

## 3 Similarities and differences in pharmacological experiments

The introduction of quinoline heterocycles, and the insertion of rigid heterocycles between the A and B rings seem to be a common interest of all major researchers in all designed quinoline compounds. By observing the designed compounds, it is found that the double bond is substituted with a heterocyclic ring, and the B ring is substituted with a quinoline ring to obtain a compound with higher activity, such as compound **47** (MCF-7, IC_50_ = 3 ± 2 nM). Some of them also modify the compounds designed by their predecessors, including the substitution of substituents or modification of linkage bonds, all to more active compounds, such as compound **53** and **57**, which had mentioned above.

**FIGURE 13:**
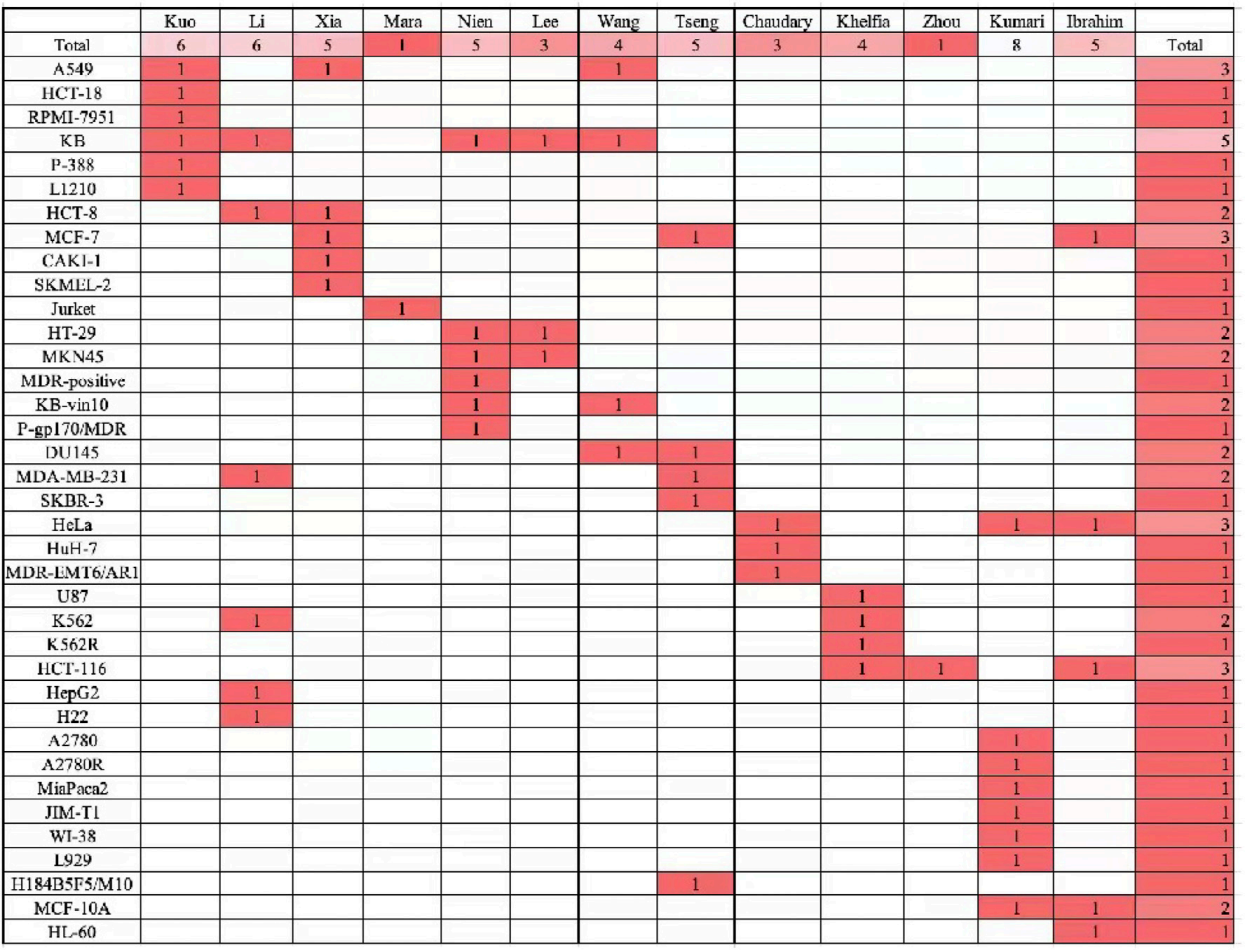
The researchers used all cell lines.

Among the compounds they designed, the human cancer cells they used the most were KB, which was used by five investigators, followed by MCF-7, HCT-116, and HeLa, which were used by three investigators each (Figure 13). Of course, after the antiproliferative activity assay in the human cancer cells they selected, they continued to select the compound with the best activity for the next mechanistic study. Their mechanistic studies included cell cycle assays, immunofluorescence assays, apoptosis assays, microtubulin polymerization inhibition assays, competitive colchicine binding assays, mitochondrial machine reactive oxygen mechanism assays, Western blot assays, cell scratching assays, and cell colony assays as well as molecular docking assays. In their pharmacological experiments, tubulin polymerization inhibition experiments were involved, indicating that this pharmacological mechanism experiment is an important means to prove the effective activity of the compounds. Some experiments have explored deeper pharmacological mechanisms, such as EB1 gene transfection and EB1 comet count, as well as the alteration of a series of apoptosis induced after SAG activation, such as compound **47**.

In most cell cycle experiments, the compound with the best quinoline derivative activity blocked the selected cancer cells in the G2/M phase in a concentration-dependent manner. Similarly in apoptosis experiments, the percentage of apoptotic cells increases with increasing compound concentration. In immunofluorescence experiments, the microtubule state disrupted by the compounds is usually seen, even with multinucleation. In microtubule protein polymerization inhibition assays, most quinoline compounds exhibit inhibition of microtubule proteins, some even beyond CA-4. In mitochondrial mechanism assays, compounds cause a collapse of the mitochondrial membrane potential, which leads to apoptosis. In Western blot experiments, compounds triggered apoptosis in cancer cells by downregulating the expression level of the anti-apoptotic protein Bcl-2 and upregulating the expression of the pro-apoptotic protein Bax, while activating caspase-9 also triggered apoptosis. Cell scratching experiments also illustrated that quinoline-like microtubulin inhibitors can inhibit the migration of cancer cells. For the selected derivatives with activity beyond CA-4, the researchers further conducted animal experiments, taking subcutaneous inoculation of cancer cells and observing the size of tumor cells in mice after drug treatment, as well as observing changes in body weight of mice, and they found that most of the drugs with good activity had a manageable effect on body weight of mice. These pharmacological experiments can provide strong evidence for the follow-up research on these drugs.

## 4 Concluding remarks

This paper reviews all the designed and synthesized CA-4 analogues containing quinoline structures from 1992 to 2022, from the beginning of design to synthesis to pharmacological activity studies. Among these numerous quinoline-based derivatives, several good compounds deserve further push, such as compound **46**, which has been studied in detail from design to synthesis and then *in vitro*, and the results of the study are in line with expectations and are ready to be studied for consideration to enter the clinical study part. In addition, we did statistics on quinoline derivatives and found that the most used cell in the antiproliferative activity assay was the KB. Therefore, we suggest that the future design of quinoline derivatives can be considered to use this cell for the study. One point worth mentioning is that various researchers have only made modifications to the structure and stability, including modifications to the A and B ring, insertion of rigid thickened heterocyclic quinolines or indoles between the A and B ring, and substitution of 3,4,5-trimethoxyphenyl for other heterocycles. Fortunately, the modified derivatives have maintained better activity, and some even surpassed CA-4. However, for the modified toxicity of the compounds, only three investigators have conducted experiments on two non-human cells and confirmed the high selectivity of their designed compounds. Therefore, we suggest that the selectivity for normal cells can be improved on the existing basis. We encourage other researchers to also select potent compounds from mechanistic studies beyond the other mechanistic pathways studied above, not limited to apoptosis. For example, we propose the use of potent compounds in combination with cell signaling pathways for deeper studies. Finally, these quinoline-containing derivatives enrich the diversity of tubulin inhibitors, and also lay a foundation for the druggability of these compounds to go to the clinic.

## Glossary

CA-4: Combretastatin A-4
CBSI: Colchicine binding site inhibitor
A-549: Human lung carcinoma cell lines
HCT-8: Ileocecal carcinoma cell lines
RPMI-7951: Melanoma cell lines
KB: Oral epidermoid carcinoma cells
P-388 and L1210: Two murine leukaemia cell lines
MCF-7: Breast cancer cell lines
CAKI-1: Renal cancer cell lines
SKMEL-2: Melanoma cancer cell lines
ROS: Reactive oxygen species
PRAP: Poly ADP-ribose polymerase
HT-29: Human colorectal carcinoma cell lines
MKN45: Human gastric cancer cell lines
H460: Non-small-cell lung carcinoma cell lines
MDR: Multiple drug resistance
MRP: Multi-drug resistant protein
KB-vin: P-gp-expressing multidrug-resistant cell lines (vincristine-resistant KB)
DU145: Prostate cancer cell lines
H184B5F5/M10: Non-cancerous normal epithelial cell lines
MDA-MB-231: Breast cancer cell lines
SKBR-3: Breast cancer cell lines
CIL-102: A known inhibitor of tubulin polymerization
HuH-7: Hepatocellular carcinoma cell lines
EMT6/AR1: Drug-resistant mouse breast cancer cell lines
U87: Human astrocytoma cell lines
K562: Acute phase chronic myeloid leukaemia cell lines
HCT-116: Human colon cancer cells
A27280: Human ovarian cancer cell lines
HepG2: Human hepatocellular carcinomas cell lines
H22: Mouse hepatocellular carcinoma cell lines
HUVECs: Human umbilical vein endothelial cell lines
A2780R: Cisplatin-resistant human ovarian tumor cell lines
MiaPaca2: Human pancreatic cancer cell lines
JIM-T1: Breast cancer cell lines
L929: Fibroblasts cell lines
MCF-10A: Non-tumorigenic mammary epithelial cell lines
CI: Chromosome consistency index
SAC: Spindle assembly checkpoint
