# Supplementary material for: Advances in antitumor research of CA-4 analogs carrying quinoline scaffold
Source: Front Chem. 2022 Oct 28;10:1040333. doi: 10.3389/fchem.2022.1040333 (PMC9650302; doi:10.3389/fchem.2022.1040333)
Supplement: Supplementary file 1 [file DataSheet1.PDF]

Number of structures	Cell lines	IC_{50}	References
Compounds **23,25,26**	A-549, HCT-8, RPMI-7951, KB, P-388 and L1210	EC_{50} < 1.0 μg/mL	(Kuo et al., 1993)
Compounds **21, 23, 24, 27-29, 33, 40**, and **41**	NCI-H226, DMS 114, HCT-116, KM20L2, OVCAR-3, RXF-393, SK-Mel-5, SF-268 and SF-295	GI_{50} micromolar to nanomolar range	(Li et al., 1994b)
Compounds **43**, and **44**, **45**	HCT-8, MCF-7, A-549, KB, CAKI-1 and SKMEL-2	ED_{50} nanomolar or sub nanomolar range	(Xia et al., 1998)
Compound **47**	Jurkat cells	GI_{50} 0.45 ± 0.10 μM	(Ferlin et al., 2010)
Compound **48**	KB, H460, HT29, and MKN45	0.2 - 0.7 nM	(Nien et al., 2010)
Compounds **49**	KB, HT29 and MKN45	mean 42 nM	(Lee et al., 2011)
Compounds **50**	KB, HT29 and MKN45	mean 12 nM	(Lee et al., 2011)
Compound **51**	KB, HT29, and MKN45	mean 30 nM	(Lee et al., 2012)
Compound **52**	KB, HT29, and MKN45	mean 57 nM	(Lee et al., 2012)
Compound **53**	KB-vin	GI_{50} 1.5 - 1.7 nM	(Wang et al., 2013b)
Compounds **54, 55, 56** and **57**	A549, KB, KB-vin and DU145	GI_{50} 0.011 - 0.19 μM	(Wang et al., 2013b)
Compound **58**	MDA-MB-231	0.75 μM	(Tseng et al., 2015)
Compound **59**	MCF-7	3 ± 2 nM	(Chaudhary et al., 2016)
Compound **60**	A549	< 10 nM	(Khelifi et al., 2017)
Compound **61**	MCF-7	1.5 ± 0.7 nM	(Zhou et al., 2017)
Compound **62, 63**	K562	2 ± 1 nM	(Li et al., 2019)
Compound **64**	HCT-116	70 pM	(Khelifi et al., 2019)
Compound **65**	MCF-7	0.026 ± 0.002 μM	(Ibrahim et al., 2020)
Compound **66**	MCF-7	0.010 ± 0.003 μM	(Ibrahim et al., 2021)
